# The Epidemiology of Malignant Melanoma during the First Two Years of the COVID-19 Pandemic: A Systematic Review

**DOI:** 10.3390/ijerph20010305

**Published:** 2022-12-25

**Authors:** Ana-Olivia Toma, Mihaela Prodan, Akash Reddy Reddyreddy, Edward Seclaman, Zorin Crainiceanu, Vlad Bloanca, Felix Bratosin, Catalin Dumitru, Ciprian Nicolae Pilut, Satish Alambaram, Neeharika Gayatri Vasamsetti, Luminita Decean, Marius Pricop

**Affiliations:** 1 Department of Microbiology, “Victor Babes” University of Medicine and Pharmacy, Eftimie Murgu Square 2, 300041 Timisoara, Romania; 2 Department of Plastic Surgery, “Victor Babes” University of Medicine and Pharmacy, Eftimie Murgu Square 2, 300041 Timisoara, Romania; 3 Doctoral School, “Victor Babes” University of Medicine and Pharmacy, Eftimie Murgu Square 2, 300041 Timisoara, Romania; 4 School of General Medicine, Bhaskar Medical College, Amdapur Road 156-162, Hyderabad 500075, India; 5 Department of Biochemistry and Pharmacology, “Victor Babes” University of Medicine and Pharmacy, Eftimie Murgu Square 2, 300041 Timisoara, Romania; 6 Center for Complex Networks Science, “Victor Babes” University of Medicine and Pharmacy, Eftimie Murgu Square 2, 300041 Timisoara, Romania; 7 Methodological and Infectious Diseases Research Center, Department of Infectious Diseases, “Victor Babes” University of Medicine and Pharmacy, 300041 Timisoara, Romania; 8 Department of Obstetrics and Gynecology, “Victor Babes” University of Medicine and Pharmacy, 300041 Timisoara, Romania; 9 Kaloji Narayana Rao University of Health Sciences, Nizampura, Warangal, Telangana 506007, India; 10 Faculty of General Medicine, George Emil Palade University of Medicine, Pharmacy, Science and Technology, Strada Gheorghe Marinescu 38, 540139 Targu Mures, Romania; 11 Discipline of Oral and Maxillo-Facial Surgery, Faculty of Dental Medicine, “Victor Babes” University of Medicine and Pharmacy, Eftimie Murgu Square 2, 300041 Timisoara, Romania

**Keywords:** COVID-19, SARS-CoV-2, oncology, malignant melanoma, skin cancer

## Abstract

It is hypothesized that the COVID-19 pandemic had a major impact on the epidemiology of malignant melanoma owing to diminished screening, diagnostic, and treatment capacities, resulting in a more advanced stage at initial presentation. The goal of this study is to undertake a systematic analysis of all epidemiological and clinical data on the trends and patient outcomes with malignant melanoma during the ongoing pandemic. Records were identified from PubMed, Cochrane, and Web of Science, selecting a total of 39 articles, narrative reviews, and editorial letters, following the PRISMA guidelines. The vast majority of the studies were published in Europe (28/39), and North America (7/39). A total of 99,860 patients were analyzed during 2020 and 2021 of the COVID-19 pandemic, and it was observed that malignant melanoma TNM staging increased significantly compared to the pre-pandemic period. Before the pandemic, 25.88% of patients had TNM stage II or above, compared to 36.25% during 2020–2021. During the COVID-19 pandemic, the malignant melanoma Breslow depth index grew from 1.59 mm before 2020 to 1.86 mm in 2020 and 2021. Patients decreased by 19.58% in 2020 and 2021 compared to pre-pandemic numbers. The patient–loss ratio indicated lower screening activity and patient addressability to dermatology and plastic surgery departments with skin cancer concerns during the COVID-19 pandemic. This systematic study shows that the identification and management of malignant melanoma during the COVID-19 pandemic faced major challenges which should alert medical systems to the high number of patients with advanced disease stages who may need emergency treatment and become incurable.

## 1. Introduction

After more than two years since the beginning of the COVID-19 (coronavirus disease, 2019) pandemic have passed, it is undoubtable that the pandemic had a significant impact on the global population and medical systems [1,2]. After March 2020, the World Health Organization (WHO) labeled the severe acute respiratory syndrome COVID-19 outbreak a worldwide pandemic [3]. Consequently, the majority of European nations and many countries around the globe implemented a near-total lockdown in an attempt to prevent the SARS-CoV-2 spread in the population and successfully assisted national health systems [4,5]. Consequently, planned procedures ceased, preserving important medical assets, expanding the number of ICU beds, and keeping patients and hospital staff from catching the illness. This phase also limited the availability of medical services, delayed commonly recognized critical procedures, and discouraged patients from seeking therapy [6,7].

Throughout this period, disturbances in medical services have prompted concerns regarding potential delays in the management of skin cancer, particularly concerning malignant melanoma that has a worse prognosis [8,9]. Data on tumor development models to predict the impact of diagnostic delays due to lockdown on the size of malignant melanoma tumors revealed a considerable rise in the percentage of tumors with a worse outcome due to advanced stage of disease and diagnosis [8,10].

We hypothesize that the COVID-19 pandemic has had a significant effect on malignant melanoma epidemiology due to decreased screening and diagnosis and treatment capabilities, determining a higher staging at diagnosis of malignant melanoma [11,12]. With almost three years having passed since the beginning of the pandemic and the increased availability of long-term data, we attempt to conduct a comprehensive evaluation in a systematic review of all epidemiological and clinical research on malignant melanoma trends and patient outcomes. 

## 2. Materials and Methods

### 2.1. Study Design

This systematic review was registered in the PROSPERO database for systematic review methodologies [13] and adhered to the Preferred Reporting Items for Systematic reviews and Meta-Analyses (PRISMA) recommendations [14] to provide an extensive overview of the epidemiology of malignant melanoma during the first two years of the COVID-19 pandemic. 

An extensive query was performed on PubMed, Scopus, and Cochrane Library using the terms “melanoma,” “malignant melanoma,” “COVID-19,” and “SARS-CoV-2” as subject identifiers. From March 2020 to September 2022, we examined data from the literature published as reviews, original articles, and letters to the editor, resulting in 39 qualifying papers. After reading the abstracts, EndNote was used to reject 235 papers and delete 28 duplicates. After further reading the remaining research, only papers written in English or Romanian were considered, leading to the exclusion of an additional 62 publications. In the end, 39 papers were selected for analysis. This systematic review sought to address the following questions:

*Question 1: What percentage of malignant melanoma screenings and treatments were delayed during the COVID-19 pandemic?*

*Question 2: What is the degree of change in the malignant melanoma stage at diagnosis and Breslow depth index during the COVID-19 pandemic?*

*Question 3: Are there significant differences in short-term outcomes of patients with malignant melanoma during the COVID-19 pandemic?*

### 2.2. Selection Criteria

The following inclusion criteria were used for the publications obtained from the search queries: (1) full-text, original work accepted for publication in a peer-reviewed journal; (2) only studies reporting pre-pandemic data compared to the COVID-19 pandemic period were considered for inclusion; (3) articles featuring malignant melanoma staging; (4) articles describing patients’ disease-free survival or mortality as outcomes; (5) articles describing screening or treatment delay for malignant melanoma; and (6) articles must have been written in English or Romanian language. Publications that reported non-melanoma skin cancer epidemiology and outcomes during the COVID-19 pandemic were excluded from the study. In addition, the search was confined to academic research papers; hence, book chapters, editorials, and case reports were removed.

### 2.3. Quality Assessment

Following the NHLBI-published Study Quality Assessment Tools, two researchers evaluated information from existing articles and reported results individually. The tools are unique to research designs and screen for any methodological or operational problems. The Quality Assessment Tool for Observational Cohort and Cross-Sectional Investigations was used for the remaining studies [15]. For each of the 14 questions for study evaluation, “Yes” replies were worth 1 point, while “No” and “Other” responses were worth 0 points. The final quality score was then calculated. Therefore, investigations with a rating from 0 to 4 were deemed to be of low quality, research with a grade between 5 and 9 was deemed to be of acceptable quality, and investigations with a score of 10 or more were deemed to be of good quality.

### 2.4. Data Extraction

**Figure 1:**
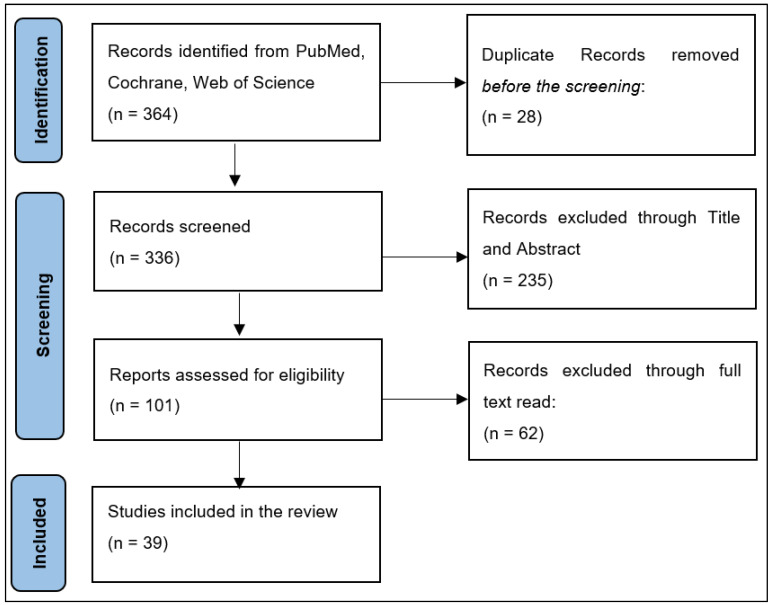
PRISMA flowchart for the study selection process.

According to our inclusion and exclusion criteria, each title and abstract were examined by two researchers in an independent manner. Any discrepancies between the two leading researchers throughout the screening procedure were handled by discussion or consultation with a third senior investigator. If uncertainty remained, the piece was included in the collection for complete perusal. The country of study, number of patients, patient demographics (age, gender), malignant melanoma staging and grading before and during the COVID-19 pandemic, the Breslow index, observed screening and treatment delays, disease-free survival, recurrence rate, and mortality rates were extracted from the papers. All information was gathered from the articles’ texts, tables, figures, and online supplemental resources. The selection procedure included eliminating duplicate entries, abstract screening, and full-text screening based on the qualifying criteria specified. Initial results from the search returned 364 entries, of which 28 were duplicates. Figure 1 shows the 39 papers included in the systematic review after abstract and title screening eliminated 235 studies, while the full-text reading eliminated 62 studies. Search query: “((COVID-19) OR (SARS-COV-2)) AND ((melanoma) OR (skin cancer)) AND (pandemic)”.

## 3. Results

### 3.1. Study Characteristics 

At the end of the study selection process, a total of thirty-nine publications were included in the final analysis, comprising thirty retrospective cohort studies, two narrative reviews, and seven editorial letters describing entirely or partially the epidemiology of malignant melanoma during the COVID-19 pandemic, in comparison with the similar pre-pandemic period. The vast majority of studies were published in Europe (28/39) and North America (6 in the US and 1 in Canada), respectively, with two published in South America (1 in Brazil and 1 in Chile), and two in Australia. The researchers reported data from 2020 and 2021 in comparison with the similar period from 2019 or earlier, insisting more on the lockdown periods from 2020 with the highest restrictions. Although a total of 99,860 patients were analyzed in the thirty-nine studies, a third of them scored poorly in quality assessment, as is seen in Table 1. 

### 3.2. COVID-19 Pandemic Effects on Malignant Melanoma 

**Figure 2:**
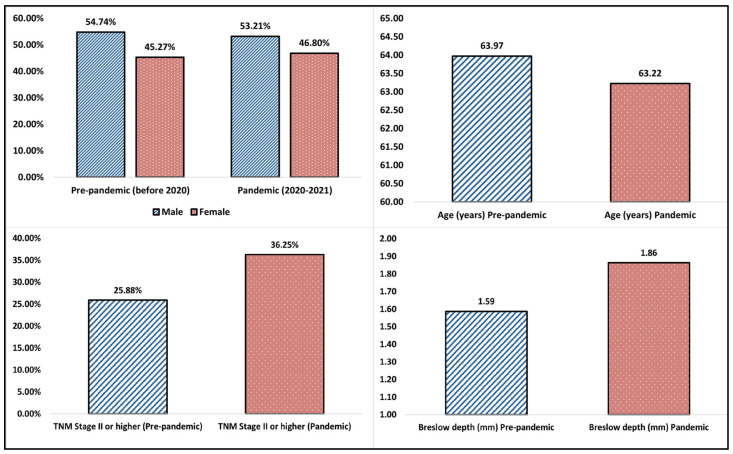
Summary of findings.

Table 2 and Table 3 describe the demographic data extracted from the studies comparing the pre-pandemic period with the COVID-19 pandemic period, as well as the epidemiology of malignant melanoma during these two periods. The majority of patients during both reported periods were men (54.74% during the pre-pandemic period vs. 53.21% during 2020 and 2021), as is seen in Figure 2. The highest proportion of male patients was observed in an American retrospective study by Davis et al. [28] from 2022, who reported a 62.9% proportion of men with malignant melanoma, followed by another American study by Lamm et al. with a 62.7% proportion of men with malignant melanoma [39]. 

Regarding the age of patients, the average age reported among the thirty-nine studies was 63.97 years in the pre-pandemic period, compared to 63.22 years during 2020–2021, without a statistically significant difference. The highest age difference was observed in a Spanish retrospective study by Martinez-Lopez et al. from 2022 [21], describing a median age of 77 years in the pre-pandemic period compared to 53 years during 2020 and 2021. This difference is likely attributed to the elderly patients’ reluctance to attend hospital visits due to the risk of a SARS-CoV-2 infection with a higher severity in this population group. On the opposite side, the lowest average age of patients with malignant melanoma was reported in a study from Chile [51], with a total of 296 patients in which the mean age was 52.7 years before the pandemic and 53.3 years during the COVID-19 pandemic.

One of the most important findings of this systematic review was the significant increase in malignant melanoma TNM staging during the COVID-19 pandemic period of 2020 and 2021. As seen in Figure 2, the average proportion of patients with a TNM stage II or higher before the pandemic was 25.88%, compared to 36.25% during the 2020-2021 period. Among the analyzed studies, the highest increase was reported by Martinez-Lopez et al. in 2022 [21], from 22.1% vs. 55.5%; however, the authors analyzed a shorter period overlapped by the pandemic lockdown. Other highly increased proportions in malignant melanoma staging were reported by Davis et al. [28] (7.1% vs. 27.5%), Hazzaa et al. [12] (58.3% vs. 79.7%), and Barcaui et al. [47] (31.3% vs. 75.0%).

Consequent to the increased TNM staging, the Breslow depth index of malignant melanoma was also significantly increased during the studied COVID-19 pandemic period, rising from an average of 1.59 mm before 2020 to 1.86 mm during 2020 and 2021. The highest increase in depth was reported in a narrative review by Cariti et al. [17], identifying a Breslow index of 0.80 mm before the pandemic, compared to 1.56 mm during the pandemic (although the patient cohort was only 172 cases). Another significant increase was observed in a study from the U.S.A. by Weston et al, with 0.78 mm before 2020, compared to 2.04 mm during 2020 and 2021. However, the total number of patients was not reported. In contrast, other studies did not find any differences in the Breslow index [37,40,48,50], and few of them even described a decrease in the depth of malignant melanoma during the COVID-19 pandemic [9,41].

The epidemiology data of malignant melanoma patients before and during the COVID-19 pandemic is described in Table 3. The patient–loss ratio was an important indicator of decreased screening activity and decreased patient addressability towards dermatology and plastic surgery departments with concerns for skin cancer during the pandemic. The average decrease in patients during 2020 and 2021 was 19.58% from a similar period before the COVID-19 pandemic, while the highest decrease was described by Cocuz et al. from Romania [24] with a 75.0% drop in the number of malignant melanoma cases during the first lockdown period from February to May 2020. Another significant decrease in patients was described in Spain [20], Berry [29], and Koch [51] (−58.8%, −48.0%, and −45.0%, respectively). However, a few studies reported an increase in the number of patients during the pandemic period (2020–2021), describing a 19.6% and 12.0% increase, respectively [9,39].

## 4. Discussion

### 4.1. Decreased Patient Presentation 

In our analysis, we noticed a rise in the Breslow depth of melanomas diagnosed in 2020 and 2021 relative to the preceding period. Furthermore, we have seen an increase in specific features associated with a poor prognosis such as treatment delay, a decline in screening and follow-up procedures, and a rise in malignant melanoma with a TNM staging higher than SII when compared to the time before the pandemic.

Although this systematic review described a significant decrease in the number of patients with malignant melanoma during the COVID-19 pandemic, the epidemiology was different in other countries such as the Netherlands. This research on a late diagnosis of melanoma found only a modest shift toward unfavorable melanoma stages during the first lockdown in the Netherlands but no effect in subsequent time periods [52]. This is a surprising conclusion, as a negative impact of a delayed diagnosis was anticipated beforehand. Although a rise in melanoma diagnoses was noted after the first lockdown, not all undetected melanomas had been identified within the time span of this investigation. Given the long follow-up period of >1 year after the first lockdown, it is unlikely that many severe skin tumors remain unnoticed [53]. In addition, a slower-than-previously-assumed growth rate of melanomas might account for the limited effect on tumor features. Typically, studies documenting tumor development rates of these skin cancer kinds are based on patients’ recollections of when they first saw a skin lesion, when they first considered it worrisome, and when they were diagnosed or removed [54]. Nevertheless, the variable capacity of subjects to recollect these dates precisely may reduce the validity of these investigations. On the basis of these investigations, however, prediction models indicating an unfavorable influence on tumor dimensions and prognosis owing to postponed diagnosis were developed.

The exposure of nursing staff, health care providers, and patients to asymptomatic SARS-CoV-2 brings significant danger, and the postponement of visits for dermatology patients with fever or respiratory problems was inadequate to stop the progression of the pandemic [55]. A significant decline in consultations was observed in both private clinics and healthcare facilities. In ambulatory care, the frequency of dermatologic examinations was reduced, and primarily individuals with suspected or confirmed malignancy or those receiving biologic medications were allowed visits. In some developed countries, patients with chronic skin disease were given the choice of uploading clinical pictures and/or receiving a video assessment [56].

It is vital to limit the nosocomial spread from asymptomatic, infected people because skin lesions may play a role in indirect viral transmission. To minimize patient flow and avoid congestion in outpatient dermatological departments, labs, surgical units, and medical facilities adopted extraordinary steps [57]. It was suggested that outpatient appointments for non-acute skin problems and cosmetic operations be delayed. To reduce the risk of disease dissemination by droplets, screening operations in sexually transmitted illness clinics were halted, and only clinical trial visits were planned. Furthermore, extending dermatologists’ working hours and assigning them to shifts prevented overpopulation [58].

One study revealed that only around 30% of appointments during the lockdown period needed in-person visits, with 11% requiring urgent action; the remaining patients were consulted through teledermatology [59]. Another survey conducted in March 2020 indicated that just half of the dermatological clinics were open, 31% of clinics only saw emergency patients, and the remainder were closed [60]. From the third week of February to the third week of March, the average number of patients seen every week in the United States decreased by almost fifty percent, according to a web-based poll. Two-thirds of respondents anticipated a further decline of more than fifty percent in the coming weeks. Approximately two-thirds of non-essential consultations were postponed throughout this time period [61,62,63].

Throughout the COVID-19 pandemic, studies reported a proportionally similar distribution of malignant skin lesions, with a non-significant decrease in malignant melanoma in sun-exposed areas such as the face and neck to a more increased distribution on the trunk and extremities, compared to the pre-pandemic period where the face and neck were more commonly involved. Additionally, a significant decline in the incidence of minor operations, such as biopsy, cryotherapy, and electrosurgery, was observed. In a U.S.A study, the frequency of weekly biopsies plummeted from twenty to more than fifty percent throughout the outbreak’s onset and during lockdown periods [64]. The removal of benign tumors, such as cysts and lipomas, and aesthetic operations were postponed. Improving infection prevention strategies during dermatologic surgery is explored further in this article. Dermatologists needed to modify their strategies for eliminating skin cancer, concentrating on the diagnosis of melanoma and removing high-risk lesions while delaying treatments for low-risk malignancies. In the care of skin cancer, intermediate-risk tumors were examined on a case-by-case basis and often delayed [65].

### 4.2. Increased Staging and Grading

The COVID-19 pandemic has also altered the existence of high-risk and poor prognostic indicators for melanoma advancement, such as Breslow thickness and advanced staging. Similar investigations on the influence of the pandemic on clinical and pathologic melanoma features have been reported by several studies in multiple European countries and the U.S.A [21]. A recent Italian multicenter study analyzed melanomas removed within two months after the restrictions were lifted and found an increase in Breslow thickness, frequency of ulcerated melanomas, and the number of mitoses, particularly in melanomas identified in northern Italy [27]. Some regions, such as Milan, Italy, were considered to be the epicenter of COVID-19 in Europe during the first waves of the pandemic, where the healthcare systems were overwhelmed by the number of SARS-CoV-2 infections and therefore only the most urgent consultations were allowed, affecting the dermatology practice as well (as reported by Giacalone et al. [65]). Similar findings were registered in most of the countries that adopted strict lockdown measurements during the first year of the pandemic. In our review, we detected the majority of studies reporting an increase in Breslow thickness, the number of mitoses, and the proportion of ulcerated melanomas compared to the year before the pandemic.

In one recent study of the Austrian population, no variations in Breslow were identified one year after childbirth; nevertheless, statistically significant differences in the occurrence of ulceration were observed. Other investigations conducted on the Spanish and American populations have shown a considerable rise in Breslow thickness after the outbreak [37,66]. In both trials, a rise in the diagnosis of thick melanomas was also seen. In addition, one American research study indicated an increase in mitoses and satellites over the time analyzed after the COVID-19 pandemic was declared. Possible differences in the statistics provided in various nations may be attributable to the non-uniform amplitude of pandemic waves over the globe, and the results may be impacted by the time studied in each series. During the pandemic waves, a study from the Netherlands revealed a considerable change in the observed and expected melanoma patients compared to pre-pandemic periods [37]. The poorer tumor staging of individuals identified after the commencement of the pandemic was also a result of their late melanoma diagnosis. The decrease in the proportion of in situ melanoma diagnoses and the rise in invasive melanoma diagnoses seen in our analysis is consistent with what the majority of authors have reported. However, one study that covered a longer period in analyzing the epidemiology of skin cancer found that in the past fifteen years—from 2006 to 2020, including the pandemic period—the proportion of invasive and non-invasive cases of melanoma did not differ significantly, even though there was a 12.7% in the number of new diagnoses during the first year of the COVID-19 pandemic [67].

The rise in negative prognostic factors for melanoma following the onset of the SARS-CoV-2 pandemic has led to a significant increase in the diagnosis of locoregionally advanced melanomas stage II and higher, either via a positive sentinel lymph node biopsy or via the diagnosis of lymph node metastases, in transit or satellites [31]. Thus, in our analysis, we identified a considerable rise in the incidence of melanomas detected at stage III in the post-COVID era, consistent with the findings of other researchers.

Melanoma patients diagnosed after the universal quarantine imposed by the pandemic should be predicted to have a worse prognosis in light of these factors. We detected a statistically significant decrease in the anticipated 5- and 10-year survival rates of patients diagnosed after March 2020 [23]. This may be owing to the deterioration of the histological features of melanomas as a result of delayed detection and treatment. Thus, some Spanish writers have calculated a 2% loss in 5-year survival for melanomas identified with a delay of three months or more [10].

Multiple clinical findings indicate that COVID-19 lockdown intervals have disrupted skin cancer treatment. Throughout the lockdown, the number of skin cancers identified and managed has decreased significantly. A large prospective analysis of more than 2000 patients in the United Kingdom revealed an average 30% weekly decline in the incidence of skin cancer diagnosis during the COVID-19 lockdown time [68]. Similarly, another study discovered an almost 70% drop in skin cancer diagnoses in the United Kingdom relative to the preceding year [69]. Although many Italian studies reported on the epidemiology of malignant melanoma during the COVID-19 pandemic, a particular study from Italy found no decline in the overall number of skin cancers identified between May and November 2020 but a considerable rise in the number of invasive skin cancers, including malignant melanoma, due to imposed restrictions and the fear of contracting the SARS-CoV-2 infection [45,70]. In this investigation, invasive skin malignancies were characterized as melanomas of stages T1b and above. Therefore, throughout pandemics, it is crucial to maintain skin-referral networks.

One U.S study found that the average monthly number of skin cancer diagnoses reduced dramatically during peak pandemic months, matching lockdown times, with just a slight rise throughout the recovery phase beginning in the summer of 2020 [71]. During the lockout time, the number of malignant melanomas diagnosed in the United States decreased by over 50 percent, according to a retrospective chart analysis. The authors also hypothesized that the backlog of undetected tumors through the recovery period of June to August 2020 might result in diagnostic difficulties of one to three months on average for melanomas.

### 4.3. Management of Malignant Melanoma during the COVID-19 Pandemic 

Regarding the third question raised by this systematic review, asking if there are significant differences in short-term outcomes of patients with malignant melanoma during the COVID-19 pandemic, due to poor follow-up, data on skin cancer outcomes like disease-free survival (DFS) and mortality during COVID-19 are inadequate. Similarly, only four studies provided the DFS of recently diagnosed MM patients in our study. It has been shown that little delays in cancer treatment may have a major impact on long-term survival. Recent model-based assessments of cancer outcomes influenced by the pandemic revealed a loss of 1–2 life-years per person with surgical delays of 3 and 6 months for all malignancies [63,72]. 

In a poll conducted in the United Kingdom, over half of Mohs surgeons reported suspending treatment during lockdowns due to redirected resources, lack of personal protective equipment, or fears of virus transmission [73]. In Italy, Filoni and colleagues were surprised to find that surgical excisions increased by more than 30%, whereas sentinel lymph node biopsies and lymph node resections decreased by 29% and 64%, respectively [16]. The rise in surgical excisions may be linked to the reallocation of people from elective operations to oncologic consultation channels.

The National Comprehensive Cancer Network recommends delaying treatment of malignant melanoma in situ for up to three months if necessary during the pandemic. T1 stages may also be postponed for up to 3 months, even if the biopsy margin is positive, as long as the bulk of the lesion has been excised [74]. Additionally, extensive surgery may be postponed for up to three months for invasive melanoma of any depth for whom a prior biopsy revealed clean histologic margins or just peripheral in situ component involvement. Priority should be given to the surgical treatment of T3/T4 (>2 mm thick) melanomas over 2 mm in thickness. The biopsy of sentinel lymph nodes may be postponed for up to three months unless a large excision is planned, in which case both operations might be done simultaneously. As seen in our review, several studies reported a delay in sentinel lymph node biopsy, lymph node dissection, and follow-up [75].

Moreover, immunosuppressive medications used for malignant melanoma have not been linked to an elevation in pulmonary infection caused by COVID-19. Furthermore, it was documented that three immunosuppressant-treated toddlers tested positive for COVID-19 but had only minor symptoms and no respiratory problems [76]. Patients who test positive for COVID-19 should undertake an interdisciplinary risk assessment prior to discontinuing immunomodulators because of the potential for withdrawal adverse effects. Currently, available evidence suggests that immunosuppressants are safe for patients during the COVID-19 pandemic. Despite the paucity of evidence, there is unanimity that individuals who require systemic medication and have no COVID-19 symptoms may resume medication [77]. 

### 4.4. Study Limitations 

Although there were many eligible studies that met the inclusion criteria for this systematic review, the reported information was, in many cases, incomplete, or the reported data did not follow the same measurement and categories, making it difficult to summarize the findings, such as the precise staging of malignant melanoma. Moreover, there was a high heterogeneity of data and variables. Thus, few studies were given a good quality assessment score. Nevertheless, only a few studies outside Europe and USA reported pandemic data about the epidemiology of malignant melanoma, so it is difficult to generalize these findings.

## 5. Conclusions

As has been demonstrated by the findings of this systematic review, the detection and management of malignant melanoma during the SARS-CoV-2 pandemic encountered significant obstacles, which should raise awareness for medical systems as they will be confronted with a large number of patients with advanced disease stages who may require emergency treatment and may become incurable in later stages. Generally, it was observed that a significantly higher TNM stage and Breslow depth index followed the patients with malignant melanoma identified during the COVID-19 pandemic period. Consequently, it is vital to conduct urgent and effective measures to balance the decrease in patients during the pandemic period and avoid the decline in malignant melanoma screening and treatment.

## Figures and Tables

**Table 1:** Studies included in the analysis.

No.	First Author (Year)	Country	Study Type	No. of Patients	Comparison Date	Quality Assessment
1 [16]	Filoni (2020)	Italy	Retrospective	NR	23 February 2020–21 May 2020	Poor
2 [17]	Cariti (2021)	Italy	Review	172	May–June (2017 + 2018 + 2019 vs. 2020)	Moderate
3 [18]	Wong (2020)	England	Retrospective	2759	January–June (2019 vs. 2020)	Good
4 [19]	Dika (2021)	Italy	Retrospective	562	January–December (2019 vs. 2020)	Poor
5 [20]	Tejera-Vaquerizo (2021)	Spain	Retrospective	559	March–June (2019 vs. 2020)	Good
6 [21]	Martinez-Lopez (2022)	Spain	Retrospective	130	15 March 2019–15 March 2020	Moderate
7 [22]	Gisondi (2021)	Italy	Editorial letter	1190	March–October (2019 vs. 2020)	Moderate
8 [23]	Guven (2021)	Turkey	Retrospective	43	March–December (2019 vs. 2020)	Poor
9 [24]	Cocuz (2021)	Romania	Retrospective	50	April 2019–February 2020 vs. April 2020–February 2021	Poor
10 [25]	Pala (2020)	Italy	Retrospective	169	1 January 2020–30 April 2020	Poor
11 [26]	NR (2021)	Italy	Retrospective	11,324	February–April (2019 vs. 2020)	Poor
12 [27]	Gualdi (2021)	Italy	Retrospective	1124	1 May–31 July (2017 + 2018 + 2019 vs. 2020)	Moderate
13 [28]	Davis (2022)	USA	Retrospective	688	August 2019–March 2020; May 2020-December 2020; January–February 2020 vs. May 2020–June 2020	Good
14 [29]	Berry (2021)	Australia	Retrospective	NR	30 March–30 June 2020 vs. 1 February–27 March 2020; 2017+2018+2019 vs. 2020	Poor
15 [30]	Asai (2021)	Canada	Retrospective	595	6 January–19 April (2019 vs. 2020)	Moderate
16 [31]	Weston (2021)	USA	Retrospective	NR	June–August (2015 + 2016 + 2017 + 2018 +2019 vs. 2020)	Moderate
17 [12]	Hazzaa (2022)	Romania	Retrospective	301	January 2018–January 2020 vs. January 2020–January 2022	Good
18 [32]	Kleeman (2022)	Germany	Retrospective	61,732	18 March 2019–17 March 2020	Moderate
19 [33]	Chang (2021)	USA	Retrospective	237	March–May (2019 vs. 2020)	Moderate
20 [34]	Ricci (2022)	Italy	Review	531	1 January–9 March 2020 vs. 2021 10 March–3 May 2020 vs. 2021 4 May–6 June 2020 vs. 2021	Moderate
21 [35]	Micek (2022)	Germany	Retrospective	366	1 January 2019–1 March 2021	Good
22 [11]	Makaranka (2022)	Scotland	Retrospective	4502	2019 vs. 2020	Moderate
23 [36]	Shannon (2021)	USA	Retrospective	325	15 June–15 August (2019 vs. 2020)	Moderate
24 [37]	Hoellwerth (2021)	Austria	Retrospective	1365	February–July (2018 + 2019) vs. 2020	Moderate
25 [38]	Van Not (2022)	Netherlands	Retrospective	1318	(2018 + 2019) vs. 2020	Good
26 [39]	Lamm (2022)	USA	Retrospective	111	May 2019–September 2021	Moderate
27 [40]	Welzel (2022)	Germany	Editorial letter	940	2019 vs. 2020 vs. 2021	Moderate
28 [41]	Gedeah (2021)	Spain	Retrospective	592	(2018 + 2019) vs. 2020	Poor
29 [42]	McFeely (2021)	Ireland	Retrospective	162	2019 vs. 2020	Moderate
30 [43]	Kostner (2022)	Switzerland	Retrospective	1240	1 February 2019–30 April 2021	Moderate
31 [44]	Mollinier (2022)	France	Editorial letter	373	March–October (2019 vs. 2020)	Poor
32 [45]	Valenti (2021)	Italy	Retrospective	461	18 May–18 November (2019 vs. 2010)	Moderate
33 [46]	Trepanowski (2022)	USA	Editorial letter	3896	1 March 2019–29 February 2020 vs. 1 March 2020–28 February 2021	Poor
34 [47]	Barcaui (2022)	Brazil	Retrospective	91	January–March (2018/2019 vs. 2020/2021)	Poor
35 [9]	Seretis (2021)	Greece	Retrospective	47	20 May–20 September (2019 vs. 2021)	Moderate
36 [48]	Balakirski (2022)	Germany	Retrospective	986	2019 vs. 2020 vs. 2021	Moderate
37 [49]	Shaikh (2022)	USA	Editorial letter	492	11 March 2020–12 January 2021 vs. 1 March 2019–10 March 2020	Moderate
38 [50]	Villani (2020)	Italy	Editorial letter	131	(2018 + 2019) vs. 2020	Poor
39 [51]	Koch (2021)	Chile	Editorial letter	296	January 2019–March 2020	Poor

NR–Not reported.

**Table 2:** Demographic data extracted from the studies comparing the pre-pandemic with the pandemic period.

No.	Male %	Age *	Stage	Breslow Index (mm)
1 [16]	NR	NR	NR	NR
2 [17]	58.5% vs. 50.0%	61 vs. 55	NR	0.80 vs. 1.56
3 [18]	NR	NR	NR	NR
4 [19]	NR	NR	NR	NR
5 [20]	44.3% vs. 57.5%	64 vs. 63	>SI (25.6% vs. 34.3%)	>1 mm (34.1% vs. 44.4%)
6 [21]	55.8% vs. 43.4%	77 vs. 53	SII+SIII (22.1% vs. 55.5%)	1.08 vs. 2.65
7 [22]	55.4% vs. 56.5%	61 vs. 62	NR	>1 mm (21.0% vs. 23.0%)
8 [23]	50.5% vs. 54.4%	60 vs. 61	>SII (66.6% vs. 80.0%)	NR
9 [24]	NR	NR	NR	NR
10 [25]	60.0%	62	SIII (38.0%), SIV (62.0%)	NR
11 [26]	NR	NR	NR	NR
12 [27]	50.8% vs. 50.9%	60 vs. 59	NR	0.40 vs. 0.83
13 [28]	62.9% vs. 58.1%	65.7 vs. 67.0	SII+SIII (7.1% vs. 27.5%)	NR
14 [29]	NR	NR	NR	2.06 vs. 2.70
15 [30]	48.5% vs. 48.9%	63 vs. 63	NR	NR
16 [31]	NR	NR	>TII (2.6% vs. 9.0%) Invasive (13.8% vs. 30.0%)	0.78 vs. 2.04
17 [12]	53.4% vs. 50.7%	58.1 vs. 58.8	>SII (58.3% vs. 79.7%)	1.10 vs. 1.80
18 [32]	55.6% vs. 54.5%	NR	NR	NR
19 [33]	NR	NR	NR	NR
20 [34]	NR	NR	NR	0.88 vs. 1.40
21 [35]	57.0% vs. 63.2%	68.6 vs. 72.6	>SII (28.9% vs. 32.3%)	NR
22 [11]	NR	NR	NR	NR
23 [36]	55.8% vs. 57.5%	68 vs. 68	>SII (14.5% vs. 15.0%)	0.87 vs. 1.40
24 [37]	52.4% vs. 53.9%	60.5 vs. 63	NR	0.62 vs. 0.70
25 [38]	58.9% vs. 57.7%	67 vs. 68	>SIIIc (9.9% vs. 10.5%)	NR
26 [39]	62.7% vs. 52.5%	61.3 vs. 63.0	SI (60.8% vs. 54.1%) >SII (5.8% vs. 11.5%)	49.0% vs. 68.8% > 1 mm
27 [40]	NR	NR	SIV (19% vs. 12% vs. 21%)	1.70 vs. 1.70
28 [41]	NR	NR	NR	0.92 vs. 0.87
29 [42]	44.0% vs. 46.4%	68.5 vs. 75.5	>SII (56.3% vs. 72.6%)	1.15 vs. 1.90
30 [43]	NR	NR	NR	2.60 vs. 2.90
31 [44]	NR	NR	>SII (10.0% vs. 22.0%)	1.60 vs. 2.20
32 [45]	51.7% vs. 56.9%	64.3 vs. 65.4	>SII (1.5% vs. 2.9%)	NR
33 [46]	NR	NR	SII (14.8% vs. 18.3%)	1.49 vs. 1.77
34 [47]	NR	65.0 vs. 72.0	SII+SII (31.3% vs. 75.0%)	0.40 vs. 0.80
35 [9]	56.0% vs. 44.0%	66.2 vs. 63.4	>SII (18.2% vs. 4.0%)	6.88 vs. 1.31
36 [48]	NR	65 vs. 64	>SII (7.8% vs. 5.4%)	0.90 vs. 0.90
37 [49]	52.8% vs. 55.7%	65 vs. 65	>SII (36.1% vs. 49.2%)	1.40 vs. 2.00
38 [50]	NR	56.2 vs. 57.1	Invasive (49.4% vs. 56.0%)	4.70 vs. 4.90
39 [51]	41.9% vs. 51.4%	52.7 vs. 53.3	>SII (24.8% vs. 42.3%)	1.00 vs. 1.50

* Data reported as mean unless specified differently; NR—not reported; SI—TNM stage 1; SI—TNM stage 2; SIII—TNM stage 3; TNM—Tumor Node Metastasis cancer-staging system.

**Table 3:** Epidemiology data of malignant melanoma.

No.	Patients before/after COVID-19	Patient Ratio *	Screening/ Treatment Delay	DFS
1 [16]	NR	−3.0%	DFU (−30.2%) SFU (−37.0%) SLNB (−29.0%) SE (+31.7%)	NR
2 [17]	47 average vs. 32 (2020)	−32.0%	DFU (−20.0%)	NR
3 [18]	1294 vs. 1465	+13.0%	DFU (−31.0%)	NR
4 [19]	278 vs. 284	+2.22%	NR	NR
5 [20]	352 vs. 207	−58.8%	Excision (−41.0%)	NR
6 [21]	77 vs. 53	−18.5%	NR	94% vs. 89%
7 [22]	634 vs. 556	−12.3%	NR	NR
8 [23]	27 vs. 16	−40.7%	NR	−7%
9 [24]	40 (18.2%) vs. 10 (23.2%)	+5.0% melanoma −75.0% cases	NR	NR
10 [25]	NR	NR	TD (−29.0%)	NR
11 [26]	−31.3%	−24.4%	Biopsy (−36.5%) WLE (−22.9%) SLNB (+6.4%)	NR
12 [27]	295 average vs. 237 (2020)	−20.0%	NR	NR
13 [28]	375 vs. 313	−17.0%	Excision (−11.7%)	NR
14 [29]	NR	−48.0%	DFU (−23.0%)	NR
15 [30]	NR	NR	Excision (−27.0%)	NR
16 [31]	106 (average) vs. 102	−5.8%	NR	NR
17 [12]	163 vs. 138	−15.3%	TD (−10.8%)	76.7% vs. 65.9%
18 [32]	31,910 vs. 29822	−7.0%	Procedures (−17.0%)	NR
19 [33]	NR	NR	DFU (−23.2%) Excision (−28.1%)	NR
20 [34]	294 vs. 237	−19.4%	NR	NR
21 [35]	NR	NR	SLNB (−1.7%) DFU (−10.4%)	87.6% vs. 57.1%
22 [11]	2468 vs. 2034	−17.6%	NR	NR
23 [36]	172 vs. 153	−11.0%	NR	NR
24 [37]	466 vs. 432	−7.3%	NR	NR
25 [38]	794 vs. 524	−34.0%	NR	NR
26 [39]	51 vs. 61	+19.6%	TD (10 days)	NR
27 [40]	327 vs. 306 (average)	−6.4%	NR	NR
28 [41]	193 (average) vs. 196	+1.6%	NR	NR
29 [42]	78 vs. 84	+7.7%	NR	NR
30 [43]	NR	NR	NR	NR
31 [44]	192 vs. 181	−15.4%	NR	94.0% vs. 94.0%
32 [45]	224 vs. 237	+5.8%	SFU (+2.3 months)	NR
33 [46]	2062 vs. 1834	−11.1%	NR	NR
34 [47]	20 vs. 16	−20.0%	NR	NR
35 [9]	22 vs. 25	+12.0%	NR	NR
36 [48]	320 vs. 319 vs. 347	NR	SLNB (+9.5%)	NR
37 [49]	246 vs. 246	NR	TD (34.5 days)	NR
38 [50]	53 (average) vs. 25	−52.8%	NR	NR
39 [51]	191 vs. 105	−45.0%	NR	NR

* Patient ratio— patients before pandemic/patients during pandemic; NR—not reported; DFU—Dermatologic follow-up; SFU—Surgical follow-up; SLNB—Sentinel lymph-node biopsy; LND—Lymph node dissection; DFS—Disease-free survival; TD—Treatment delay; WLE—Wide local excision.

## Data Availability

Not applicable.
